# Intelligence

**Published:** 1807-06

**Authors:** 


### INTELLIGENCE.

Mr. Brewster, of Edinburgh, has invented a new astrometer, for finding the rising and setting of the stars and planets, and position in the heavens, which is said to be more simple in its construction, and more extensive in its application, than any before invented. The use of this instrument is thus described: To find the name of any particular star that is observed in the heavens, place the astrometer due north and south, and when the star is near the horizon, shift the moveable index till the two sights point to the star. The side of the index will then point out, on the exterior circle, the star's amplitude. With this amplitude enter the the third scale from the centre, and find the declination of the star in the second circle. Shift the moveable horary circle, till the time at which the observation is made, be opposite the star's declination, and the index will point to the time at which it passes the meridian. The difference between the time of the stars southing, and twelve o'clock at noon, converted into degrees of the equator, and added to the right ascension of the star, comes to the meridian after the sun; but substracted from it, if the star souths before the sun, will give the right ascension of the star. With the right ascensions and declinations thus found, enter a table of the right ascensions and declinations of the principal fixed stars, and you will discover the name of the star which corresponds with these numbers. The astrometer may be employed in the solution of various other problems.

Mr. George Field has invented an improved stove for heating rooms, or drying different articles, which unites the various advantages of heating, boiling, steaming, evaporating, drying, ventilating, &c. The height of the stove is about five feet and a half, its diameter two and a half, and that of the flues four inches. The external part is constructed of brick, and the internal parts of thin Ryegate or fire stone, except the top of the fire place, which is a plate of cast iron. This stove might be adapted to the drying of malt and hops, perhaps of herbs, corn, and seeds, generally. It might also be accommodated to the purposes of sugar-bakers, connected with the great fires employed for their boilers.

A Swedish naturalist has discovered the smallest animal of the order of mammalia that has been yet seen; he calls this animal Sorex Caniculatus; it is a kind of earth mouse.

Mr. Hausman has given an account of the manner in which the solution of indigo is prepared by means of an alkaline solution of red arsenic, for the use of calico printers. He merely makes a caustic alkaline solution of red arsenic, to which he adds, while it is in a boiling state, a sufficient quantity of indigo bruised, in order to obtain a very deep shade, which may be rendered more or less intense by diluting the solution of indigo with a weak ley of caustic potash.

Mr. Chaptal, who lately resigned the office of secretary of state for the home department in the French government, for the avowed purpose of devoting himself exclusively to science, has just completed a capital work, on the application of chemistry to the arts. A Translation has been undertaken in London, and will appear in the course of June.

Mr. Nicholson, to whose scientific labours this country is under so many obligations, has undertaken an entirely new Chemical Dictionary, to be printed in one large volume octavo, and it is in such forwardness that its publication may be expected in three or four months.

Dr. Adams, physician to the Small-pox Hospital, will publish in a few days, a Popular View of the present State of Knowledge in the Practice of Vaccine Inoculation.

Mr. TAUNTON, surgeon to the City and Finsbury Dispensaries, has again appealed to the public upon the necessity of establishing a fund, to be connected with charitable institutions, for the relief of the ruptured poor. He contends, that nearly one-tenth part of mankind are afflicted with hernia; of course the prevention of an evil attendant upon this calamity, is of the utmost importance. The distressing scenes which he is called on frequently to witness, and which he has described very pathetically, might, he says, generally be prevented by a proper bandage or truss, applied in the beginning of the disease, and continued with care. This might be accomplished at a small expence, compared with the good that would accrue to society; it would even be a saving to the community at large, by the prevention of accidents which always tend to increase the parochial rates.

Mr. GRIFFITH ROWLANDS, Member of the Royal College of Surgeons, London, and Senior Surgeon to the Infirmary and Lying-in Charity in Chester, will in a short time, publish a Statement of the result of Excision of the Breast, in a great number of cases of Scirrhus and Cancer. Also general Observations on the treatment of Cancer, wherein a new remedy is introduced, of very considerable efficacy in palliating that complaint.

Mr. BROOKES will commence his Summer Course of Lectures on Anatomy, Physiology, and Surgery, on Saturday, June 6, at seven o'clock in the morning, at the Theatre of Anatomy, Blenheim-street, Great Marlborough-street. The Dissecting Rooms will be open from five in the morning till four in the afternoon.

